# In-Out Surface Modification of Halloysite Nanotubes (HNTs) for *Excellent* Cure of Epoxy: Chemistry and Kinetics Modeling

**DOI:** 10.3390/nano11113078

**Published:** 2021-11-15

**Authors:** Shahab Moghari, Seyed Hassan Jafari, Mohsen Khodadadi Yazdi, Maryam Jouyandeh, Aleksander Hejna, Payam Zarrintaj, Mohammad Reza Saeb

**Affiliations:** 1 School of Chemical Engineering, College of Engineering, University of Tehran, Tehran 11155-4563, Iran; shahabmoghari42@gmail.com (S.M.); shjafari@ut.ac.ir (S.H.J.); 2 Center of Excellence in Electrochemistry, School of Chemistry, College of Science, University of Tehran, Tehran 141746-6191, Iran; m.kh.599@gmail.com; 3 Department of Polymer Technology, Gdańsk University of Technology, Narutowicza, 11/12, 80-233 Gdańsk, Poland; aleksander.hejna@pg.edu.pl; 4 School of Chemical Engineering, Oklahoma State University, 420 Engineering North, Stillwater, OK 74078, USA; payam.zarrintaj@gmail.com

**Keywords:** epoxy nanocomposite, cure kinetics, aniline, isoconversional methods, HNT

## Abstract

In-out surface modification of halloysite nanotubes (HNTs) has been successfully performed by taking advantage of 8-hydroxyquinolines in the lumen of HNTs and precisely synthesized aniline oligomers (AO) of different lengths (tri- and pentamer) anchored on the external surface of the HNTs. Several analyses, including FTIR, H-NMR, TGA, UV-visible spectroscopy, and SEM, were used to establish the nature of the HNTs’ surface engineering. Nanoparticles were incorporated into epoxy resin at 0.1 wt.% loading for investigation of the contribution of surface chemistry to epoxy cure behavior and kinetics. Nonisothermal differential scanning calorimetry (DSC) data were fed into home-written MATLAB codes, and isoconversional approaches were used to determine the apparent activation energy (*E_α_*) as a function of the extent of cure reaction (α). Compared to pristine HNTs, AO-HNTs facilitated the densification of an epoxy network. Pentamer AO-HNTs with longer arms promoted an *Excellent* cure; with an *E_α_* value that was 14% lower in the presence of this additive than for neat epoxy, demonstrating an enhanced cross-linking. The model also predicted a triplet of cure (*m*, *n*, and ln *A*) for autocatalytic reaction order, non-catalytic reaction order, and pre-exponential factor, respectively, by the Arrhenius equation. The enhanced autocatalytic reaction in AO-HNTs/epoxy was reflected in a significant rise in the value of *m,* from 0.11 to 0.28. Kinetic models reliably predict the cure footprint suggested by DSC measurements.

## 1. Introduction

Epoxy resin has a good chemical resistance, strong adhesion to metals, and low shrinkage, making it ideal for industrial applications [1,2,3]. Several approaches have been employed to enhance the anticorrosive, thermal, and mechanical performance of epoxy through the incorporation of nanoparticles as a convenient and cost-effective strategy [4,5,6,7]. For instance, a well-dispersed nanoparticle epoxy system displays good anti-corrosive resistance in its coating applications, however, surface-active nanoparticles can participate in the curing of the epoxy resin with amine curing agents, leading to higher mechanical and thermal properties [8,9,10]. Well-designed nanoparticles can boost the corrosion resistance of epoxy coatings by eliminating the diffusion rate of the corrosive agents and/or introducing anti-corrosive materials into the system [11,12]. Nanoparticles can also participate in the cure reaction of epoxy resin, which additionally supports barrier performance by providing a denser polymeric network [13,14]. The cross-linking density of epoxy coatings can even be monotonically representative of the efficiency of the modification approach [15,16]. The enhanced cross-linking density of epoxy nanocomposites with respect to blank resin is key to determining the performance of nanoparticles [15]. The cure state of the thermoset composites can be projected by the Cure Index [2].

Anti-corrosive epoxy nanocomposite coatings are a class of epoxy nanocomposites for which the surface chemistry of nanoparticles plays a key role in corrosion inhibition. Conductive polyaniline (PANI) or polypyrrole (PPy) display a redox catalytic ability at the surface of the nanoparticles, which may contribute to the formation of a passive oxide layer [17,18,19] or host an active anti-corrosion agent in their hollow lumen or pores, which can be unleashed spontaneously in stimuli-responsive corrosive media [20,21]. The encapsulation of corrosion inhibitors, such as 8-hydroxyquinolines (8-HQ), in nanocontainers is an advanced approach to eliminating the detrimental effects of the direct incorporation of a corrosion inhibitor into resin. It may interrupt the curing process, leading to early leakage of active materials and impairing the barrier properties of coatings [22].

Halloysite nanotubes (HNTs) are among several different types of nanomaterials that may be utilized as a nanocontainer in several studies, showing a high potential for controlled release purposes in smart coatings [23,24,25]. The unique characteristics of HNT, its dual surface charge in combination with a high specific surface area, biocompatibility, and low price make it a candidate for cross-linking and for the improvement in the properties of epoxy resins [26,27,28]. HNTs have often been used as a nanocontainer [29,30,31]. These nanocarriers permit a sustained release ability in aqueous environments [32,33,34]. For instance, benzimidazole-loaded HNT nanocontainers have displayed enhanced corrosion protection when incorporated into an epoxy matrix [35]. The corrosion inhibitor released at proper time actively protects the carbon steel surface. HNT nanocontainers loaded with epoxy monomers have been used to prepare a self-healing epoxy coating with good anti-corrosive properties [36]. Multilayered HNTs containing a corrosion inhibitor have been introduced into an epoxy coating. This coating displayed an improved anti-corrosive performance compared to that of neat epoxy [37]. Surface modified HNT nanocontainers at low concentrations may be dispersed uniformly in epoxy resin by conquering the van der Waals attractive forces [38]. On the other hand, it has been demonstrated that the surface chemistry of HNTs can control the cure quality for a thermoset epoxy resin through participation in the ring-opening reaction of epoxy, featured by a remarkable increase in reaction enthalpy (*ΔH_∞_*) [39]. The curing potential of HNTs modified using hyper-branched, nitrogen-rich polyethyleneimine in epoxy resin has been studied [40]. An *Excellent* cure resulted in a 31 °C increase in the glass transition temperature (*T_g_*) of the nanocomposite with respect to the *T_g_* of neat epoxy.

A novel 8-HQ-loaded HNT nanocontainer was prepared through surface modification using aniline oligomers (trimer and pentamer) and 3-(aminopropyl)triethoxysilane (APTES) coupling agent to improve the cross-linking of epoxy. Direct modification of HNTs leads to poor dispersibility in epoxy resin, which brings about several difficulties in view of the limited numbers of hydroxyl groups located on the defects and edges of the external surface [41]. An outer shell functionalized with oligoanilines induces pH sensitivity to the nanocontainers and acts as a cure facilitator due to the presence of amine functionalities in the chemical structure. Aniline oligomers acting as an outer shell possess electroactivity and redox activity. In addition, their high solubility and precise microstructure provides an opportunity to study the relationship between chain length and final properties [42,43]. Fourier transform infrared (FTIR) spectroscopy, hydrogen-nuclear magnetic resonance (^1^H-NMR) spectroscopy, and thermogravimetric analysis (TGA) techniques were used for the characterization and validation of the synthesized materials including carboxyl-capped aniline trimer (CAT) and carboxyl-capped aniline pentamer (CAP) nanocontainers. The loading of 8-HQ into the lumen of HNTs was confirmed using UV-visible spectroscopy. Scanning electron microscopy (SEM), provided from the cross-sectional area of the cryogenically fractured, fully cured epoxy composites, and energy dispersive X-ray spectrometry (EDS) were also employed to study the microstructure, morphology, and composition of HNTs and corresponding nanocomposites. The hollow and loaded nanocontainers were subsequently added to the epoxy resin in a specific concentration (0.1 wt.% of resin) to study their contribution to the curing behavior and kinetics of the epoxy matrix through differential scanning calorimetry (DSC) analysis at the different heating rates of 5, 10, 15, and 20 °C·min^−1^. A qualitative cure analysis was performed based on a general protocol using the *Cure Index* (*CI*) to label the cure state of nanocomposites as *Poor*, *Good*, and *Excellent* from variations in the enthalpy of the cure reaction (*ΔH_∞_*) and curing temperature window (*ΔT*). Additionally, a quantitative cure analysis was carried out by determining reaction kinetic models employing the isoconversional methods of Friedman and Kissinger–Akahira–Sunose (KAS). The potential of the well-synthesized nanotubes of HNT-CAP-L and HNT-CAT-L participating in the cure reaction of the epoxy resin was assessed due to the fact that they could potentially enhance the corrosion resistance of epoxy coatings by blocking the diffusion path of the corrosive agents and/or introducing anti-corrosive materials into the system. In fact, barrier performance could be viewed from the angle of formation of a denser 3D network.

## 2. Results

### 2.1. Characterization of the Synthesized Oligoanilines

**Figure 1:**
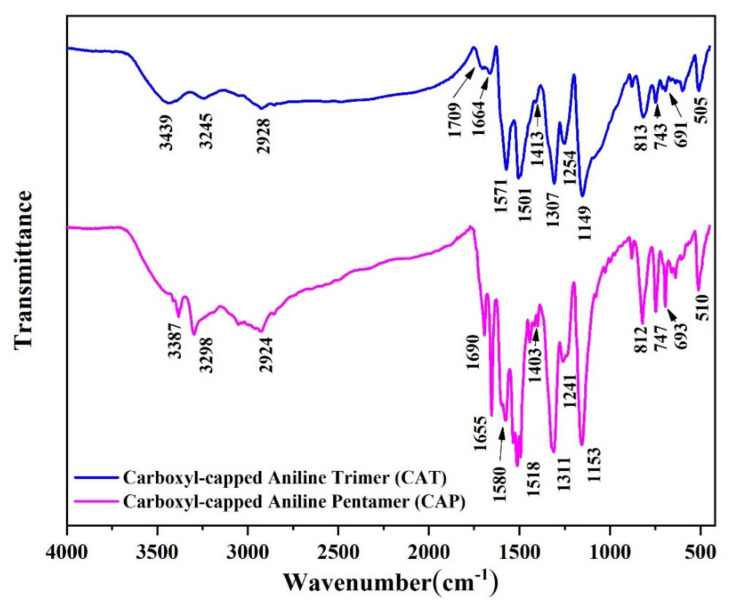
FTIR spectra of the synthesized aniline oligomers (CAT and CAP).

Figure 1 shows the FTIR spectra of the synthesized oligoanilines. The peaks appeared at around 3439 cm^−1^ in CAT, and those at 3387 cm^−1^ in CAP spectra were related to the stretching vibration of N–H functional groups and the O–H of carboxylic end-groups. The typical peaks of benzenoid (N-B-N) and quinoid (N=Q=N) rings present in the backbone of aniline derivatives were demonstrated, respectively, at 1501 and 1571 cm^−1^ in CAT, whereas those at 1518 and 1580 cm^−1^ were detected for the CAP sample [44]. There were two kinds of C=O stretching vibrations, carboxylic acid C=O and amide C=O, in the FTIR spectra of CAT and CAP, located at 1664–1709 and 1655–1690 cm^−1^, respectively. The overlap between these two peaks could result in a broader, yet less sharp, peak at 1600–1800 cm^−1^. The bands between 1650 and 1710 cm^−1^ in both structures were attributed to the stretching vibrations of carboxylic groups (C=O–O), indicating that the aniline oligomers were carboxyl-capped [45]. Moreover, the C–N stretching of the secondary aromatic amine (1307 cm^−1^ in the CAT spectrum and 1311 cm^−1^ in the CAP spectrum), together with the weaker peaks at about 1250 and 1420 cm^−1^, were indicative of the emeraldine salt state of the oligoanilines. Moreover, intensive “electronic-like” bands at 1149 and 1153 cm^−1^ in CAT and CAP oligomers, respectively, could be assigned to the vibrational mode of B–N⁺H=Q and B–N⁺H–B doped structures. The out-of-plane deformations of C–H bonds on the benzene ring (690–1150 cm^−1^) were also observed [46].

**Figure 2:**
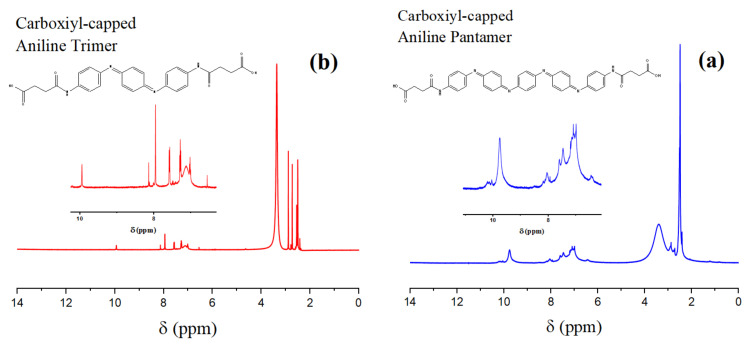
NMR spectra of the synthesized aniline oligomers (**a**) CAT and (**b**) CAP.

^1^H-NMR was additionally used for further confirmation of the structure of the oligomers (Figure 2). The characteristic peak that appeared at 2.5 ppm was attributed to the DMSO-d6 solvent. The several peaks appearing in the range of 6.5–7.6 ppm were related to the benzenoid, quinoid, and aromatic protons at different positions [47]. Moreover, the small peak at about 10 ppm was related to the NH-CO structure formed as a result of carboxyl-capping [48]. Furthermore, there were some peaks at about 2.96 and 3.33 ppm, which showed residual DMF and moisture present in the system [49].

### 2.2. Characterization of HNT Nanotubes

**Figure 3:**
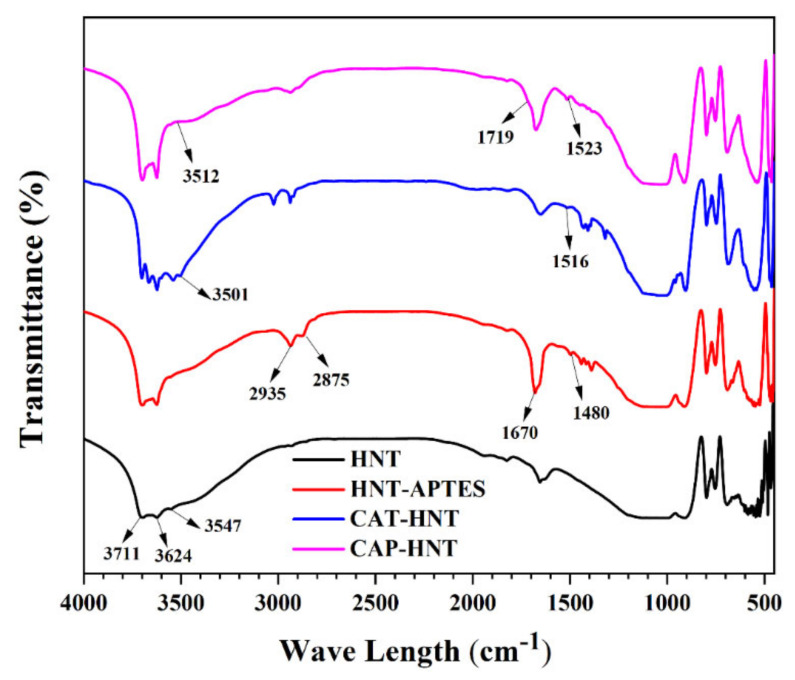
FTIR spectra of the pristine HNTs and of the HNTs surface modified with APTES, CAT, and CAP.

Figure 3 shows the FTIR spectra of the pristine and surface modified HNT. For the pristine HNTs, the characteristic peaks at 3711 and 3624 cm^−1^ were attributed to the stretching vibration of hydroxyl groups located in the lumen of nanotubes. Moreover, the peak at 3547 cm^−1^ showed the stretching of O–H bonds on the outer surface of the nanotubes [50]. The peaks related to the deformation of Al–O–Si bonds and the asymmetrical stretching vibrations of siloxane groups were observed at 539 and 1086 cm^−1^, respectively. Surface modification of HNTs with APTES introduced some small peaks at 1480 and 1670 cm^−1^, which revealed the deformation and bending vibrations of C–H_2_ and N–H_2_, respectively. In addition, the new bonds appearing at around 2875 and 2935 cm^−1^ were ascribed to the aliphatic CH_2_ bonds’ stretching vibrations. Consequently, FTIR characterization reveals the successful modification of HNTs with APTES [26].

The grafting of oligoanilines on the A-HNTs was confirmed by the peaks appearing at 3501 cm^−1^ and 3512 cm^−1^ for CAT-HNT and CAP-HNT, which were attributed to N–H stretching mode. The peaks at 1516 cm^−1^ and 1523 cm^−1^ for CAT-HNT and CAP-HNT, respectively, were related to C=C stretching vibration. The reduction in the peak intensity at 1670 cm^−^^1^ and the appearance of a small peak at 1719 cm^−1^ caused by the formation of amide groups (CONH) confirmed the reaction taking place between the carboxyl groups of oligoanilines and the amine groups of APTES present on the surface of HNTs [51]. Therefore, it can be concluded that the surface modification process performed on the halloysite nanotubes was effective and the target layers were successfully formed as shells on the surface of the nanotubes.

**Figure 4:**
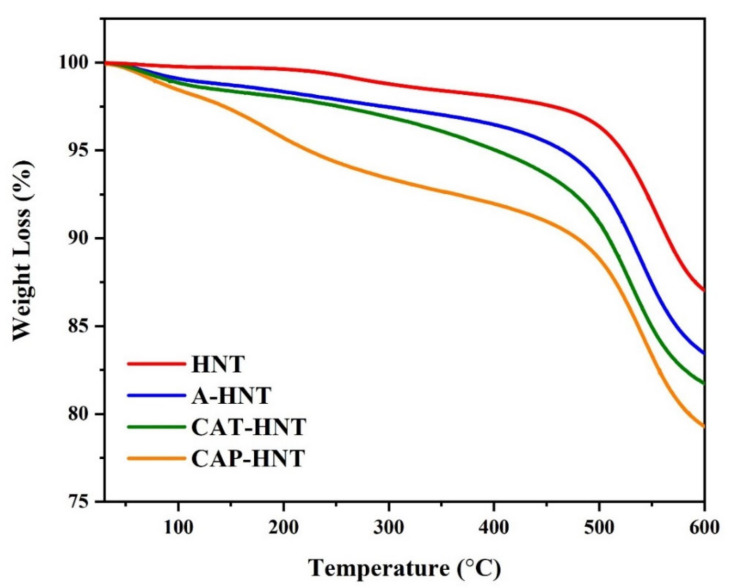
TGA thermograms of pristine HNT and of HNTs surface modified with APTES, CAT, and CAP.

The TGA thermograms of pristine and surface modified HNTs are compared in Figure 4. The slight weight loss observed at close to 100 °C was characteristic of the evaporation of the absorbed moisture. The main structural decomposition process of HNTs occurred between 300 and 600 °C when Al–OH groups underwent dehydroxylation; moreover, amino silane-grafted HNTs degraded separately as an additional decomposition step at around 250–475 °C [52]. The amount of residual char remaining from HNT degradation was 87.1%, decreasing to 85.3% for APTES-grafted HNT. As the aniline oligomers were attached to the HNTs’ surface through APTES, the amount of residual char decreased to the values of 81.8% and 79.8% for CAT- and CAP-modified HNTs, respectively. We calculated the grafting ratio using the following equation:

*G_r_* = Δ*W*/(1 − Δ*W*) (1)

where *ΔW* is representative of the mass difference between the unmodified and modified materials. Accordingly, the grafting ratio was calculated to be 3.84%, 5.59%, and 8.4% for the A-HNT, CAT-HNT, and CAP-HNT, respectively.

### 2.3. Encapsulation Confirmation

**Figure 5:**
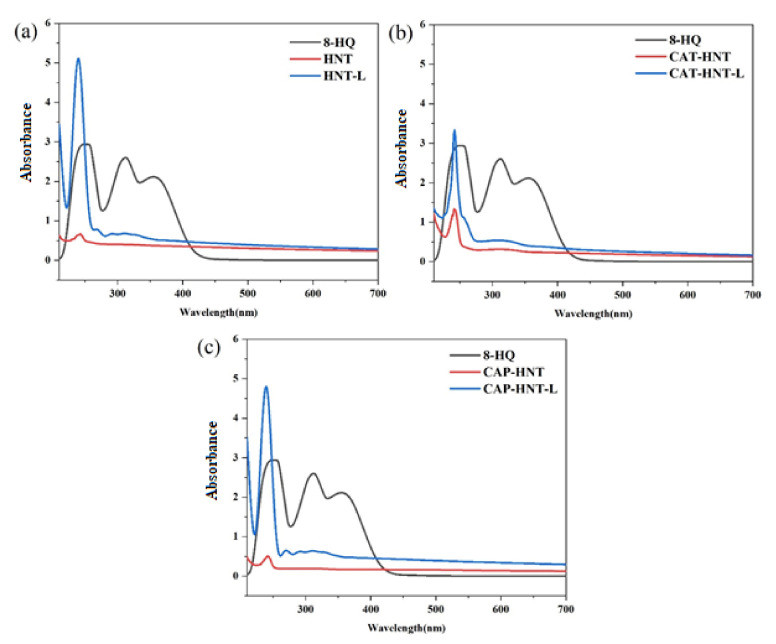
UV-visible spectra of 8-HQ, unloaded and loaded nanoparticles: (**a**) HNT, (**b**) CAT-HNT, (**c**) CAP-HNT.

UV-visible spectroscopy was used to ensure encapsulation. Indeed, 8-HQ contains a quinoline ring in its structure, which led to peaks at 243 and 318 nm, corresponding to π–π* transition [53]. The appearance of these two peaks in the UV-visible spectrum of loaded HNTs evidenced encapsulation. As can be seen in Figure 5, all the unloaded nanoparticles revealed a very slight peak at 242 nm, which was due to the colloidal clusters of HNTs formed in the solution and corresponds to π–π* transition, i.e., π electron transition from LUMO to HOMO [54]. The slight intensification of this peak in CAT- and CAP-modified HNTs can be attributed to the aromatic rings present in the oligoanilines’ structure. Evidently, the UV-visible spectrum of loaded nanocontainers was a combination of the spectra of the unloaded nanocontainers and 8-HQ, corroborating a successful encapsulation. Moreover, the color of the HNTs was altered from white to light-brown after encapsulation was completed. The CAP-modified HNTs showed a better loading efficiency compared to that of CAT-modified HNTs as they have longer chains that can effectively preserve the corrosion inhibitor within the nanocontainer.

### 2.4. Microstructure of HNTs and Epoxy/HNT-Based Nanocomposites

**Figure 6:**
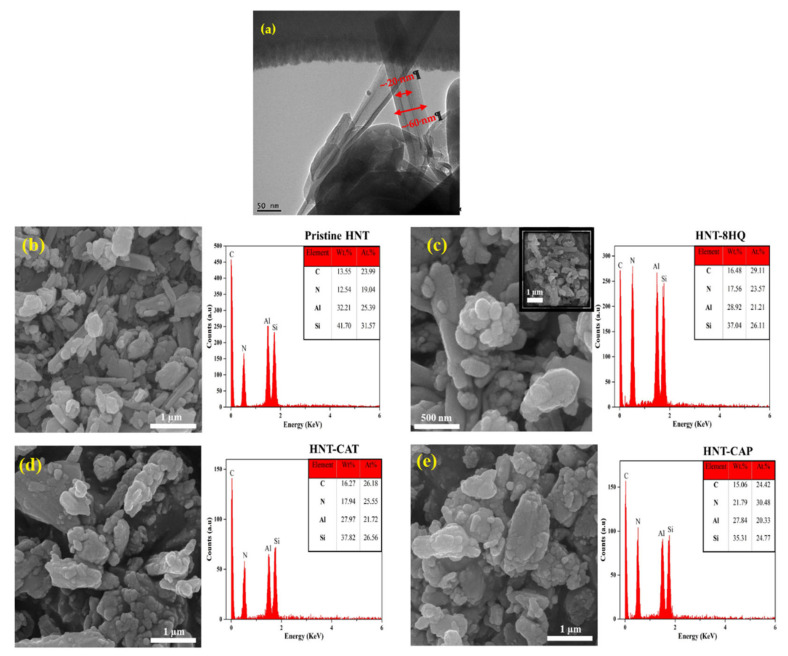
TEM images of pristine HNT (**a**), SEM and EDS of pristine HNT (**b**), 8HNT-8HQ (**c**), HNT-CAT (**d**), and HNT-CAP (**e**) nanoparticles. Red circles inside the images confirm presence of HNTs covered with aniline trimer and pentamer precursors.

The TEM micrographs of the HNT are shown in Figure 6a. In the TEM image of the HNT, the inner lumen is obviously seen due to its lighter color, with a diameter of around 20 nm and an external diameter of about 60 nm. Morphological analysis using SEM showed that the HNT tends to aggregate because of the intrinsic hydrophilic nature contributed by hydroxyl groups (Figure 6b). Once the HNT is loaded with 8-HQ, nitrogen can be better found in the microstructure, suggesting that the lumen of the HNTs is filled with 8-HQ molecules (Figure 6c). As can be seen from the SEM images of HNT-CAT and HNT-CAP, shown in Figure 6d,e, the surface of HNTs was completely covered by aniline trimer and aniline pentamer, respectively. In addition, EDS results indicated that the N percent in HNT-CAP was 21.79 wt.%, which is higher than that of HNT-CAT (17.94 wt.%); this was due to the longer chain of an aniline pentamer in comparison with an aniline trimer, which contains more amine groups.

**Figure 7:**
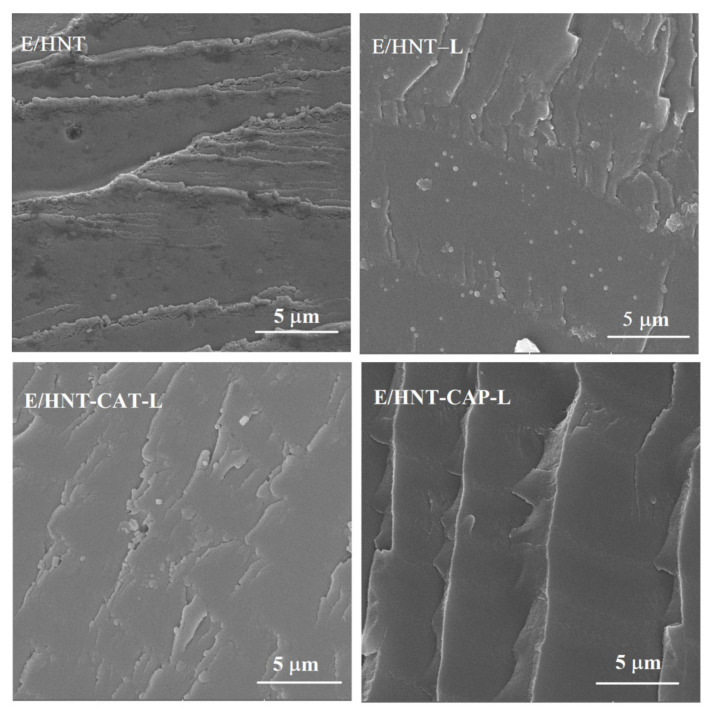
SEM micrographs of E/HNT, E/HNT-L, E/HNT-CAT-L, and E/HNT-CAP-L nanocomposites.

**Figure A1:**
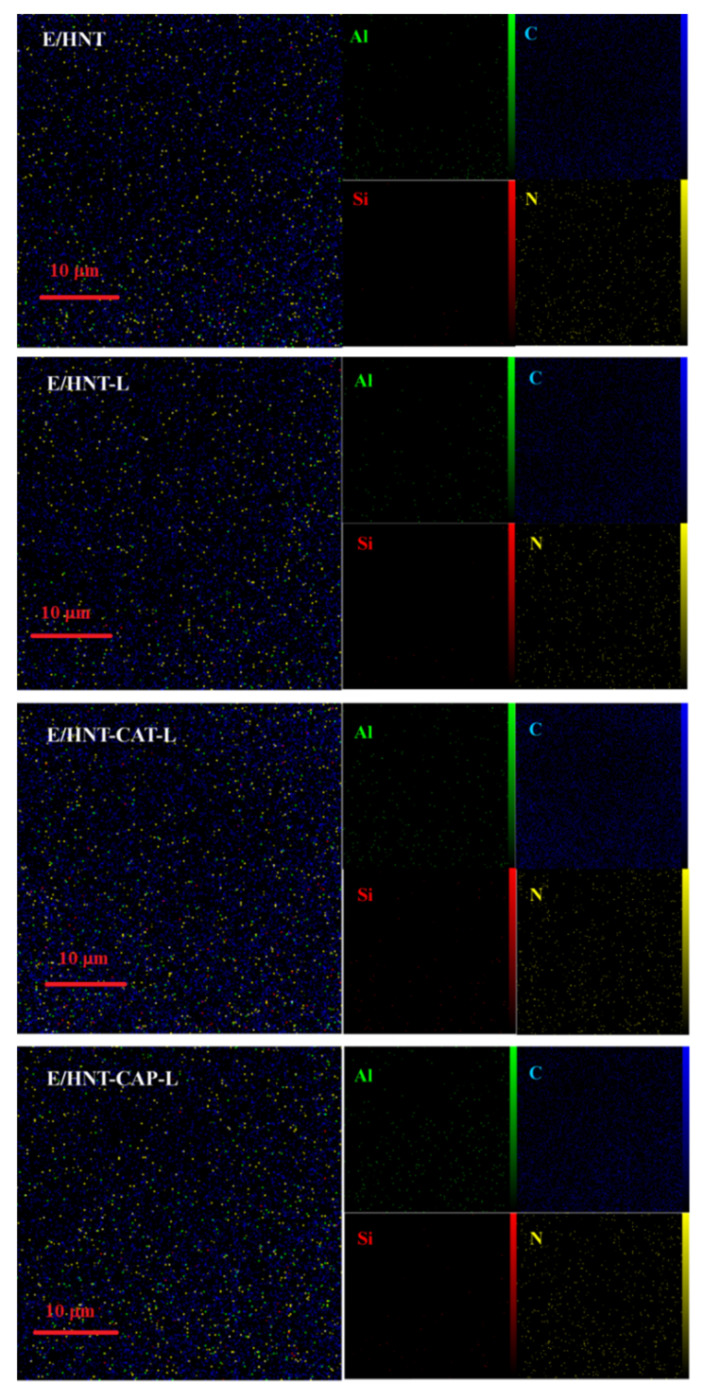
Mapping analysis of E/HNT, E/HNT-L, E/HNT-CAT-L, and E/HNT-CAP-L nanocomposites.

The morphology of the epoxy composites was investigated using SEM. As in Figure 7, several domains of aggregation were observed in the E/HNT nanocomposite, which indicated weak interfacial interaction between the epoxy and the pristine HNT. Such aggregations decrease in an HNT-L incorporated epoxy system, as obviously detected in EDS map images (Figure A1, Appendix A). Since E/HNT-CAT-L and E/HNT-CAP-L nanocomposites were denser in this network, their mapping (Figure A1) demonstrated a nano-scale distribution of the modified HNTs in the polymer matrix. As can be seen in Figure A1, Al (green dots) and Si (red dots) elements, as representatives of HNTs, were dispersed well throughout the epoxy matrix. This confirms an appropriate mixing of the nanotubes in the polymer matrix and a good compatibility between the epoxy matrix and the aniline tri- and pentamer of aniline attached to the surface of HNTs.

### 2.5. Qualitative Cure Analysis

**Figure 8:**
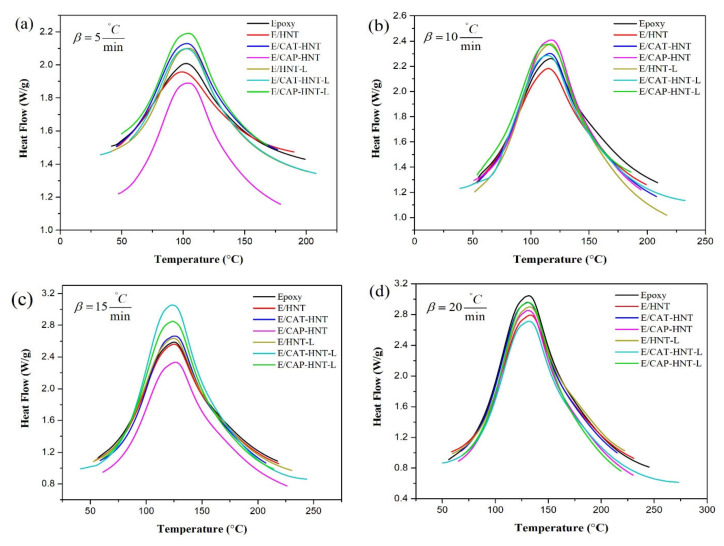
Nonisothermal DSC thermograms of epoxy and epoxy/HNT nanocomposites at heating rates of 5 (**a**), 10 (**b**), 15 (**c**), and 20 (**d**) °C·min^−1^.

Cure behavior of epoxy nanocomposites containing 0.1 wt.% of unloaded and 8-HQ-loaded pristine HNT, and CAT-, and CAP-modified HNTs were analyzed through nonisothermal DSC analyses performed at heating rates of 5, 10, 15, and 20 °C·min^−1^ (Figure 8). As can be observed, all the samples showed a similar unimodal exothermic shape over the curing temperature window (*ΔT*), regardless of the nanoparticle type. This indicates that the addition of nanoparticles to the epoxy resin did not affect the curing reaction. In other words, the formation of the epoxy network in all systems is mainly governed by the autocatalytic chemical reaction between the molecules of epoxy resin and the curing agent [55]. These curing moieties participate in an epoxy ring-opening reaction at high heating rates more efficiently. Generally, the higher the heating rate, the easier the diffusion of curing moieties into the network. At higher heating rates, more kinetic energy can be provided to the molecules, which facilitates the collision between reactants in the system [56]. However, a lack of time in high heating rates, reflected in gelation, prevents the reaction components from interaction and may confine the curability of the system [57]. The cure characteristics, including the interval of curing temperature (*ΔT*), the heat of reaction (*ΔH_∞_*), as well as the peak (T_P_), onset (*T_onset_*), and endset (*T_endset_*) temperatures of cure, are summarized in Table 1. An increase in heating rate shifted the characteristic temperatures towards higher values, a signature of the completion of cross-linking. Moreover, the introduction of pristine and modified HNTs significantly influenced the cure state of epoxy because of the different functionalities imparted to the system. Therefore, these functionalities, together with loading content and cure conditions, determine the final cross-linking state of the nanocomposites compared to the unfilled epoxy system [58]. To assess the contribution of these factors to the cure of the epoxy systems, the dimensionless *Cure Index* (*CI*) was calculated and reported based on the following equation [2]:

*CI* = Δ*H*˚ × Δ*T*˚ = (Δ*H_C_*/Δ*H_Ref_*) × (Δ*T_C_*/Δ*T_Ref_*) (2)

where the *C* and *Ref* subscripts refer to the total values of the assigned characteristics for the epoxy nanocomposites and the blank (reference) epoxy as the control sample, respectively. The constant loading content considered in this study simplified the tracking of cure behavior in nanocomposite systems.

**Figure 9:**
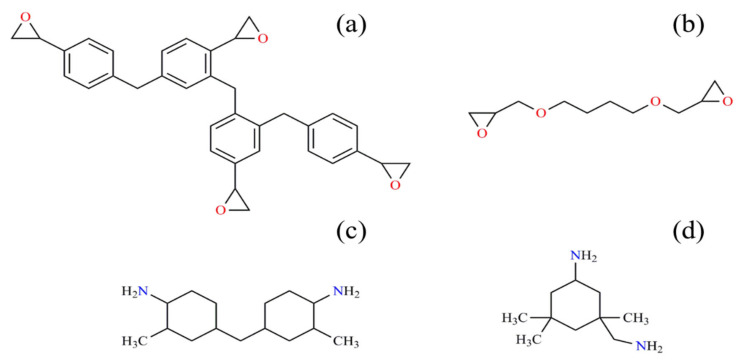
Chemical structures of Araldite LY 5052 epoxy resin components: (**a**) epoxy phenol novolac, (**b**) butanediol digylicidyl ether and Aradur HY 5052 hardener components, (**c**) cycloaliphatic diamine, and (**d**) IPDA.

Table 1 suggests that all nanocomposites containing pristine HNTs were flagged as being of a *Poor* cure state in spite of several hydroxyl functionalities present on the surface of HNTs. Therefore, these nanoparticles played an inhibiting role in the cure reaction of epoxy. Although the incorporation of HNTs in epoxy decreased *ΔT* in comparison to the neat epoxy resin (which signifies a faster reaction), it undesirably decreased the heat release during the reaction. This indicates that fewer epoxy ring-opening reactions occurred in the system. This can be attributed to the lack of interfacial compatibility between HNTs and epoxy molecules and, consequently, the aggregation of nanotubes, which implies the importance of surface modification. Moreover, about 40% of the two-component epoxy resin used in this study was epoxy phenol novolac resin (Figure 9) consisting of four aromatic rings [59]. The size of the epoxy molecules and the hydroxyl groups located on the defects and edges of the HNT’s surface [41] signifies a geometric restriction in the system that deteriorates the dispersion and accessibility of the components. The surface modification of HNTs with oligoanilines changed the cure state of epoxy to *Excellent* at low heating rates. It can be realized that the attachment of several amine functionalities to the surface of nanotubes through chains of oligoanilines improved the probability of collision in the system at low heating rates. Such a reactive-rich system provided enough time for the cure moieties to react, even after gelation. At higher heating rates, the higher kinetic energy could not compensate for the shortage of time. Furthermore, the *CI* values at all heating rates, except for 20 °C/min, were in *Good* and *Excellent* cure states as a result of the encapsulation of 8-HQ in the nanoparticles. The small molecules of 8-HQ tagged on the surface of nanotubes, but not in the lumen, could easily diffuse in the sludgy quasi-solid reaction medium and act as a plasticizer in the systems. 8-HQ could enter in-between the layers of nanotubes and polymers to react with the epoxy groups inaccessible to the other larger molecules through their amine and hydroxyl functionalities. This significantly increased the heat of reaction (heat of cure was increased by 44% for E/CAT-HNT-L sample at heating rate of 15 °C/min) but widened the curing temperature window. Therefore, the cure state changed from *Excellent* to *Good* due to how epoxy groups consumed without any network formation. Surprisingly, aniline pentamer-modified HNTs maintained their *Excellent* cure state when 8-HQ was loaded into the nanocontainers. This can be attributed to a lack of 8-HQ molecules attached to the surface of the nanotubes or the ability of aniline pentamer shells to entrap the corrosion inhibitor inside the HNT’s lumen. It can be suggested that loading efficiency as well as shell material properties play vital roles in the cure efficiency of thermoset nanocomposites containing core-shell nanocontainers. In this case, longer chains of aniline pentamer compared to aniline trimer appeared more beneficial for designing such a system. They not only facilitated the incorporation of more amine functionalities into the cure process, but also accepted more corrosion inhibitor inside the nanocontainer due to more chain entanglements.

### 2.6. Quantitative Cure Analysis

The cure behavior and kinetics of epoxy cure reaction were studied by nonisothermal DSC data at different heating rates. By assuming a direct relationship between the enthalpy of the cure and the progress of the reaction, the degree of curing conversion (*α*) at a specific temperature (*T*) was calculated as follows:

*α = ΔH_T_/ΔH_∞_* (3)

**Figure 10:**
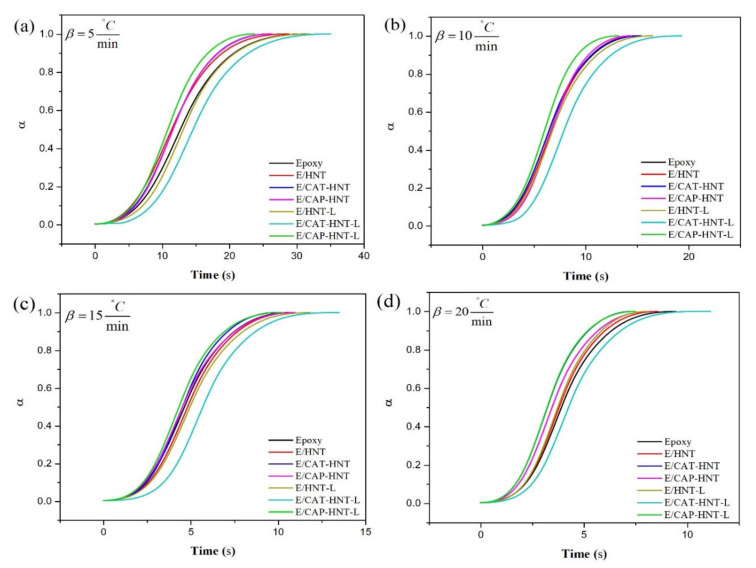
Fractional extent of conversion vs. time for epoxy and epoxy/HNT nanocomposites at heating rates of (**a**) 5, (**b**) 10, (**c**) 15, and (**d**) 20 °C min^−1^.

This equation shows the fraction of curing enthalpy up to the temperature (*T*) (*ΔH_T_*) with respect to the total heat released in the curing process (*ΔH_∞_*) [60]. The plot of fractional extent of conversion against time provides a tool to evaluate whether the incorporated nanoparticles accelerated or decelerated the curing process of epoxy resin. Figure 10 demonstrates the sigmoidal shape of α-time curves for all the samples, indicating that autocatalytic was the dominant cure reaction in the systems [61]. In such a sigmoidal plot, the initial and final stages provide the accelerating and decelerating signatures, respectively, so that in an intermediate conversion value the process rate reaches its zenith because of two opposing phenomena taking place coincidentally. At low conversions, the cure reaction advances through a chemically controlled mechanism, while by reaching the gelation point in the system, the diffusion of cure moieties is the dominant phenomenon. Finally, the curing rate is slowed down by the occurrence of vitrification (*T_cure_* = *T_g_*) in the system.

According to Figure 10, the cure reaction was completed in a shorter period by increasing the heating rate, which enhanced the kinetic energy and the mobility of molecules. Epoxy nanocomposites containing CAP-HNT-L depicted the highest curing rate regardless of the heating rates. Second fastest, the epoxy sample containing unloaded CAP-HNT showed a fast cure characteristic. On the other hand, the epoxy nanocomposite containing CAT-HNT-L was flagged as the slowest cured epoxy nanocomposite at all heating conditions as the presence of free 8-HQ molecules led to the continuation of the reaction over a longer time. These observations express the significance of surface modification of nanoparticles with a proper material (here aniline pentamer) that facilitate the network formation.

**Figure 11:**
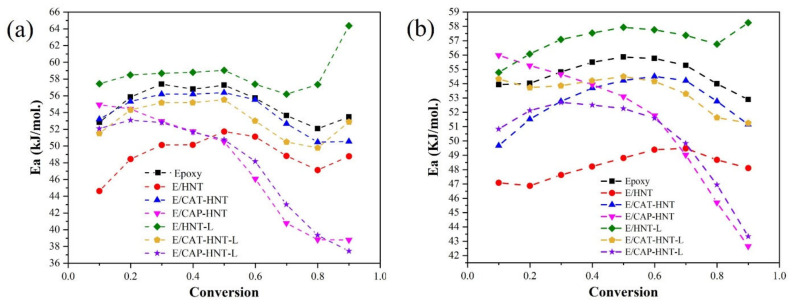
Estimation of the apparent activation energy for the neat epoxy and epoxy/HNT nanocomposites: (**a**) differential Friedman and (**b**) integral Kissinger-Akahira-Sunose (KAS) models.

**Figure A2:**
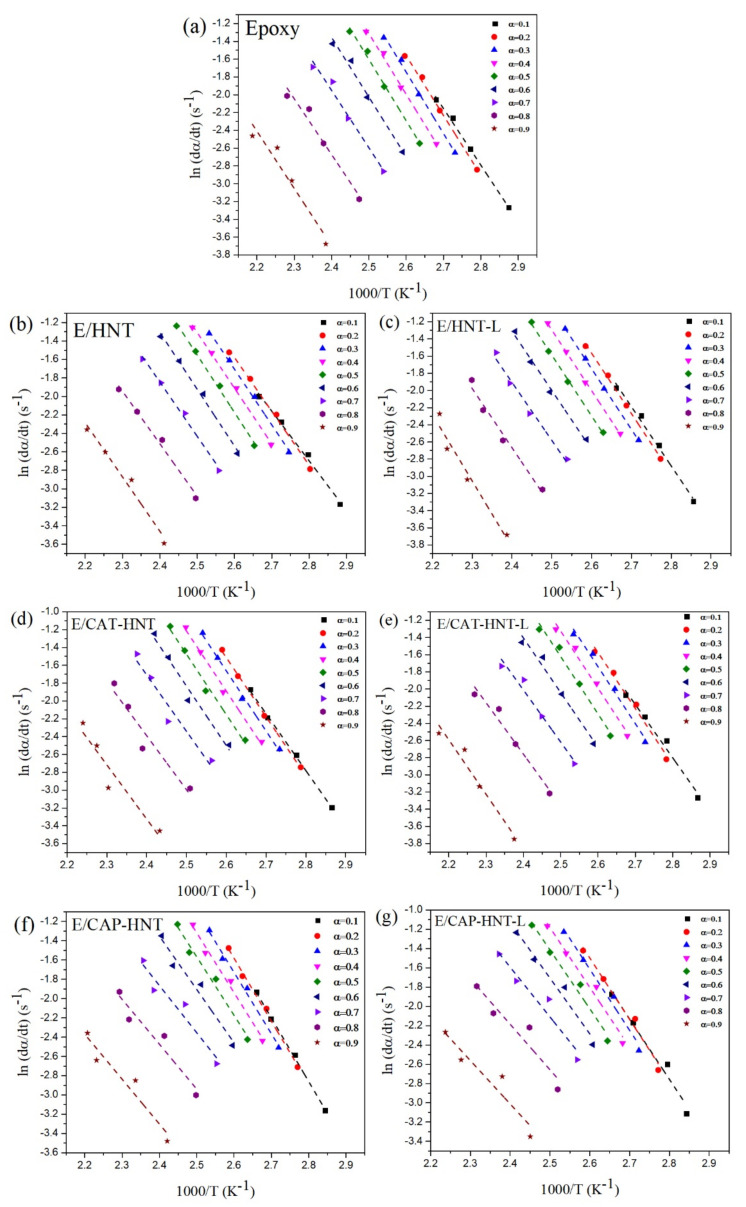
(**a**–**g**) Plots of the prepared samples based on Friedman model at *β* = 5 °C/min.

**Figure A3:**
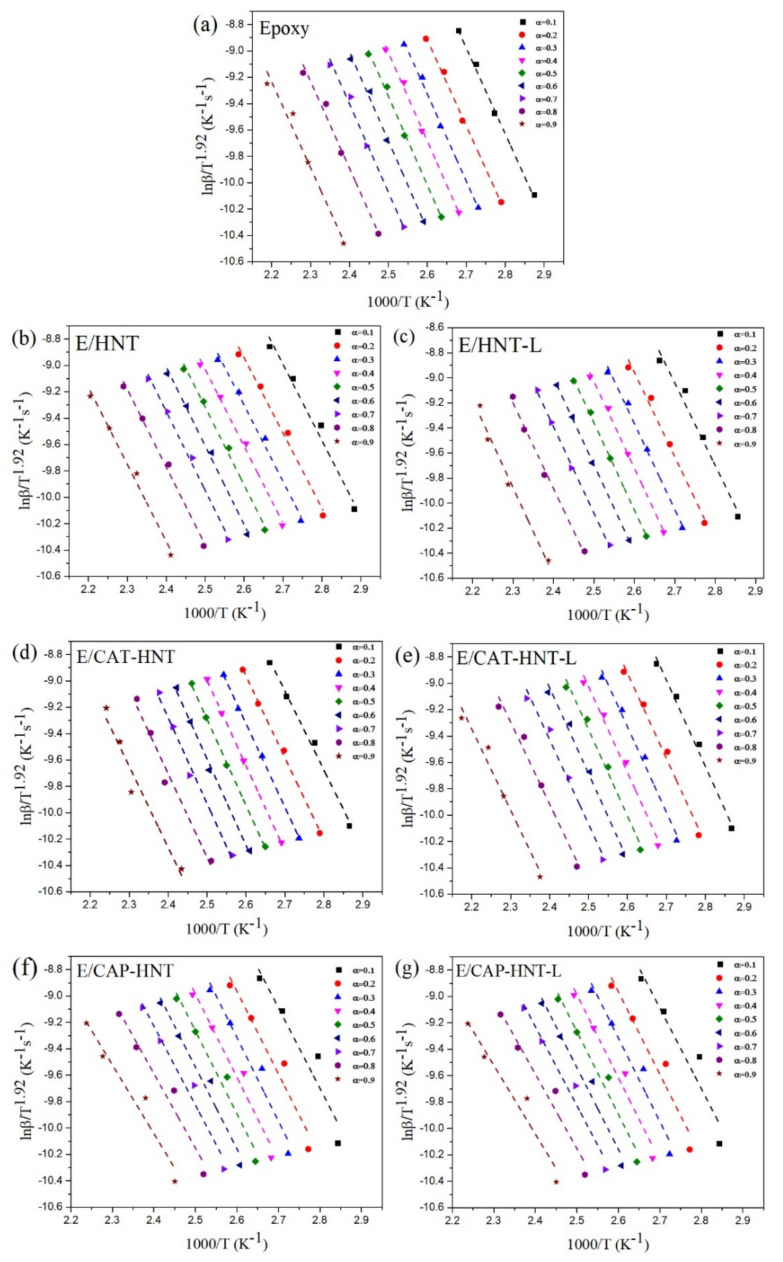
(**a**–**g**) Plots of ln(*β*/*T*^1.92^) vs. 1/*T* for prepared samples based on KAS model.

The variation of apparent activation energy as a function of the extent of cure or the conversion of the reaction were obtained for the epoxy and its nanocomposites using differential and integral model-free approaches of Friedman and KAS, respectively. The calculation procedures of apparent activation energy by both methods are described in Appendix B (Figure A2 and Figure A3) and the results are depicted in Figure 11. Approximation-free differential models are acknowledged to be more accurate than integral ones, although they introduce some inaccuracy and there are some difficulties in baseline selection, particularly for heating rate-dependent reactions [62]. In this study, both types of isoconversional methods were applied to analyze the kinetic parameters and to choose the most accurate method for cure kinetics analysis. As demonstrated in Figure 11, the apparent activation energy of neat epoxy showed a maximum value at the proximity of *α* ≈ 0.5, which was indicative of gelation. Thus, more energy for collision and the reaction of curing moieties could be considered for that system. With the addition of HNTs, a similar trend was observed while the value of *E_a_* was significantly reduced due to the functionalities introduced to the reaction medium. Although the surface modification of HNTs by CAT and CAP led to an increment in the energy required for epoxy cross-linking compared to the E/HNT nanocomposite, the curves were still located below the neat epoxy, suggesting a facilitated cure reaction. The difference in apparent activation energy values in these systems can be attributed to nanoparticle agglomeration as a consequence of the larger size of nanotubes and the lower wettability of epoxy resin. The oligoaniline chains hinder the mobility of epoxy molecules, such that entanglements of these macromolecules confine the accessibility of reactants. Interestingly, the variation behavior of *E_a_* with respect to conversion changed in the samples containing CAP-modified HNTs. The highest value of *E_a_* appeared in the early stage of reaction, resulting in the lowest apparent activation energy value among the studied systems. Accordingly, one can understand how the presence of aniline pentamer chains as a surface activators attached to the HNTs participate in the epoxy ring-opening reaction. The curing reaction is facilitated, especially at high values of conversion where diffusion governs the reaction [63]. Moreover, the encapsulation of 8-HQ corrosion inhibitor into the nanotubes did not considerably affect the *E_a_* values of the epoxy cross-linking process compared to their unloaded counterparts. However, in the case of unmodified HNTs, the encapsulation of 8-HQ resulted in the rise in *E_a_* to values higher than that of the neat epoxy sample, which can be attributed to the strong hydrogen bonding induced by 8-HQ polar molecules. This hinders the movement of the molecules in the nanocomposite system. This effect was not observed in other samples containing loaded nanocontainers because of the oligoaniline shells entrapping 8-HQ inside the nanotubes [53].

**Figure A4:**
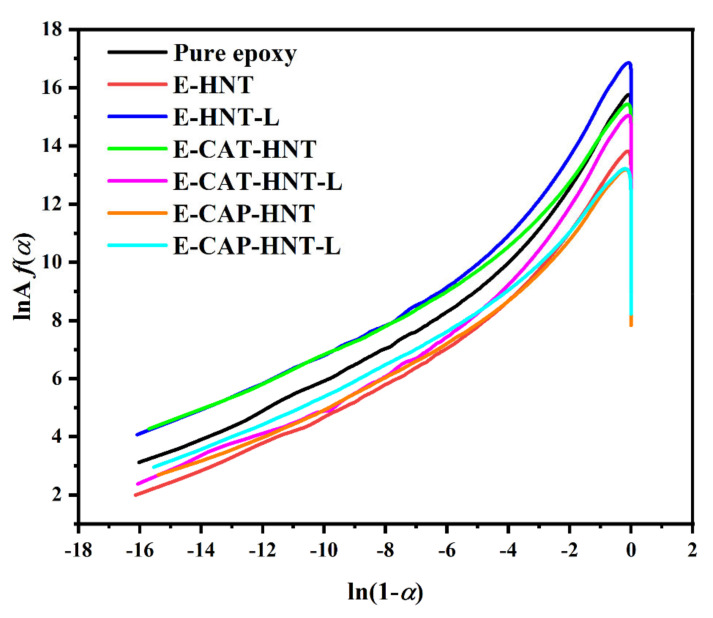
Plots of ln[*Af(α)*] vs. ln(1 *− α*) for the samples at heating rate of 5 °C/min used in calculation of apparent activation energy via Friedman method.

After calculating the value of apparent activation energy at different cure conversions, the next important step is to find the reaction model to study the role of incorporated nanoparticles in the cure reaction of epoxy systems. The mathematical relations and relevant graphs of the Friedman and Malek methods were used for the determination of non-catalytic or autocatalytic reactions, shown in Appendix C.1 and Appendix C.2, respectively. Based on Equation (A5) and the resulting curves demonstrated in Figure A4 for epoxy nanocomposite samples, one can observe that the nth-order reaction model deviates from the expected linear trend by showing a maximum value of α in the range of 0.2–0.4. The shape assigned to this curve is typical of reactions of an autocatalytic nature.

**Figure A5:**
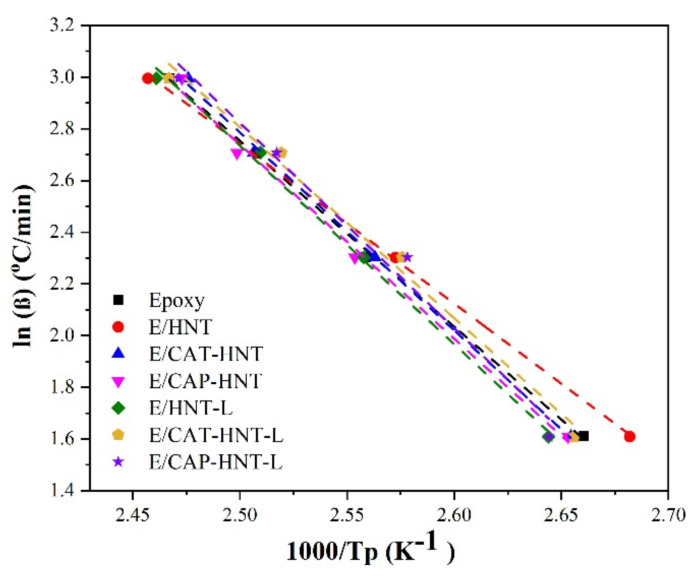
Plots of ln(*β*) vs. 1/*T* for epoxy resin and prepared nanocomposites derived from FWO model.

**Figure A6:**
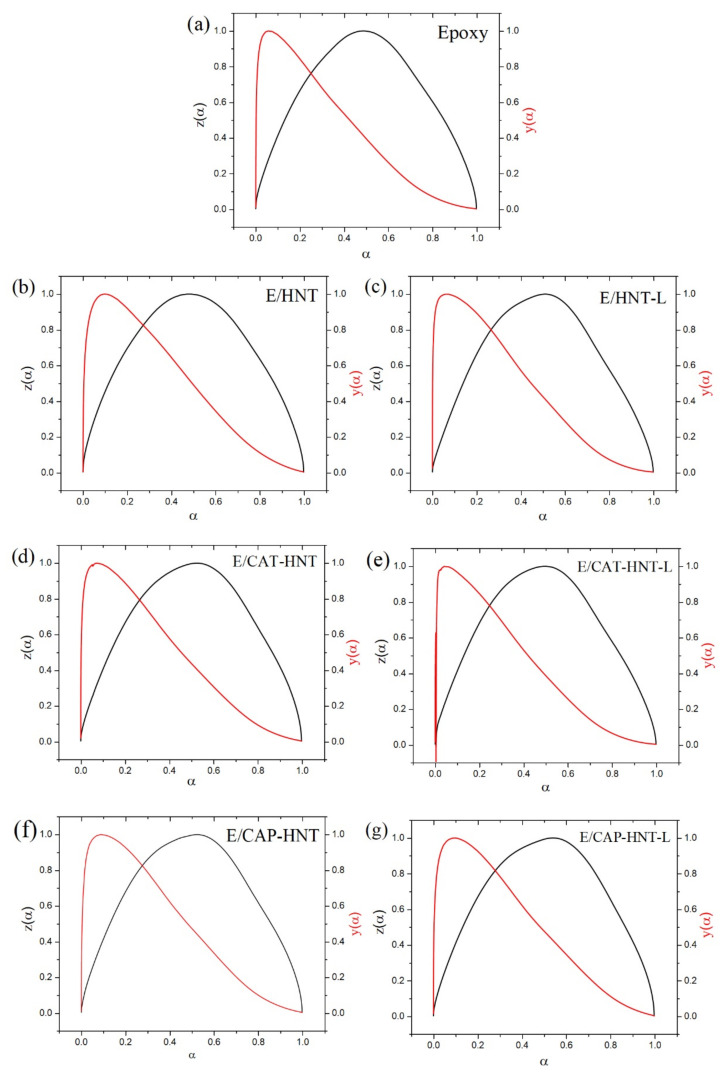
(**a**–**g**) Variation of *y(α)* and *z(α)* versus conversion for prepared samples based on Malek model.

Moreover, according to the parameters reported in Table 2 and calculated based on Malek model with constant activation energy that obtained from Flynn-Wall-Ozawa (FWO) method as shown in Figure A5. According to Malek master plots (Figure A6), the peak value of y(*α*) curves (*α_m_*) was smaller than the peak value of conversion in DSC curves (*α_p_*) at all the studied heating rates. Moreover, the peak value of z(*α*) curves (*α_p_^∞^*) possessed a value smaller than 0.632. Meeting these two criteria simultaneously suggests that a two-parameter autocatalytic reaction model should be deliberated as a kinetic model.

The truncated *Sestak*-*Berggren* equation for the autocatalytic reaction has a general form as follows:

*f*(*α*) = *α^m^* × (1 − *α*)*^n^* (4)

where *m* and *n* show the autocatalytic and non-catalytic reaction orders, respectively. This equation, together with Equation (B.2) defined in Appendix B, can be summarized to provide the rate of cure reaction:

(*dα*/*dt*) = *A* × exp(−*E_α_*/*RT*) × *α^m^* × (1 − *α*)*^n^* (5)

The value of *E_a_* has previously been calculated based on Friedman and KAS methods. Other kinetic parameters, including the frequency factor (*A*) and reaction degrees (*m* and *n*), were determined according to the mathematical and graphical descriptions provided in Appendix D, where the average amount of *E_a_* was used over the whole range of conversion. Table 3 and Table 4 represent a summary of results from the Friedman and KAS models, respectively.

Putting Table 3 and Table 4 under scrutiny, it can be realized that the values of kinetic parameters obtained by the Friedman method were very similar to the ones achieved from the KAS method. For all the samples, the degree of non-catalytic reaction was significantly higher than that of autocatalytic reactions. In addition, the overall order (*m*+*n*) of curing reaction at all the heating rates and for all the epoxy nanocomposites exceeded the value of one, which is a sign of the complexity of curing reaction. It can be observed that for all the samples except for the E/HNT-L nanocomposite, the frequency factor decreased, demonstrating the hindering effect of nanomaterials on collisions between the cure moieties. Moreover, the highest value of the autocatalytic reaction degree (up to 2.5 times greater than the neat epoxy) was attributed to the nanocomposites containing CAP modified HNTs, which reveals the contribution of CAP amine functionalities to the improvement in the autocatalytic nature of cross-linking. The introduction of loaded HNTs and CAT-modified HNTs to the system led to a drop in the autocatalytic reaction order and, consequently, a rise in the non-catalytic reaction order. Thus, it is clear that the nanomaterials used in this study were potent for changing the network formation in comparison to the neat epoxy system. The reactivity, chain length, and loading efficiency of the nanoparticles, as well as their dispersion state in the epoxy matrix, were among the most important factors affecting the cure kinetics of the prepared epoxy nanocomposites.

**Figure 12:**
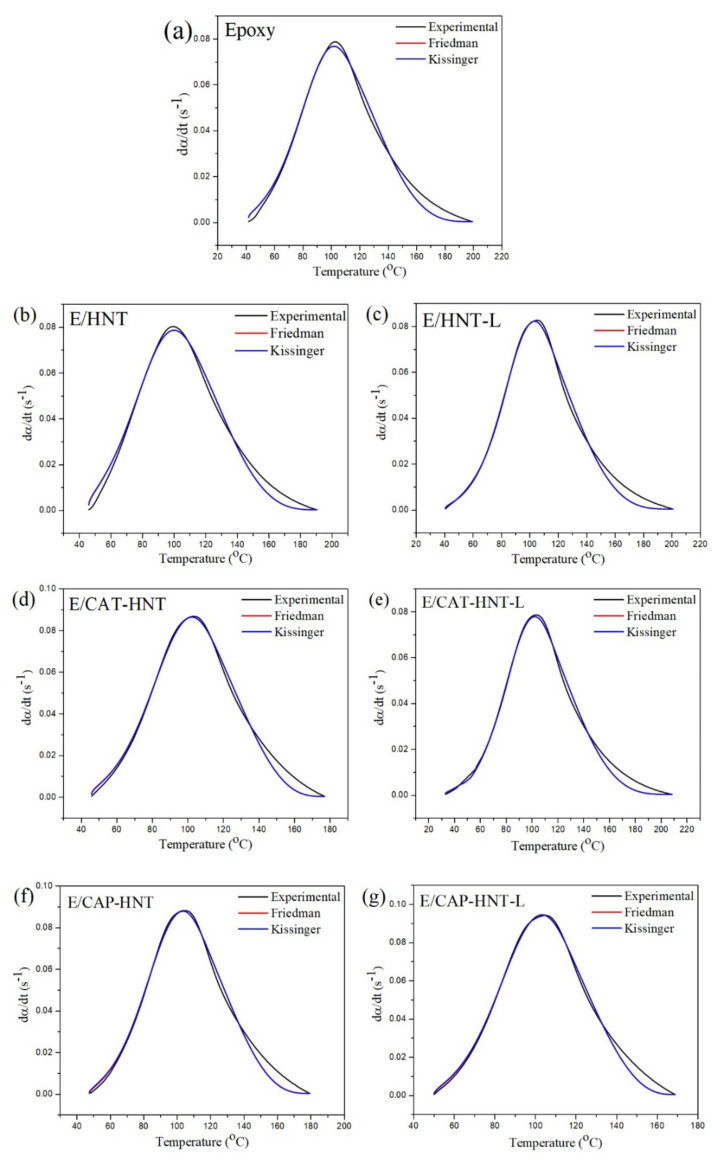
Predicted kinetics plots based on the *n*th order decomposition reaction model for neat epoxy (**a**) and its nanocomposites containing 0.1 wt.% amounts of HNT (**b**), HNT-L (**c**), CAT-HNT (**d**), CAT-HNT-L (**e**), CAP-HNT (**f**), and CAP-HNT-L (**g**), at the heating rate of 5 °C·min^−1^.

A simple and quick way to validate the resulting models and kinetic parameters is to compare the analytical curing rate plot with the experimental values. Figure 12 illustrates a good agreement between the cure kinetic models developed by the Friedman and KAS approaches with the actual values of the rate of reaction. However, slight deviations can be observed in the late stage of the curing reaction for all the samples. This can be associated with the rise in viscosity due to the occurrence of gelation and vitrification phenomena at the final phase of cross-linking reaction, when the molecular-level diffusion is of great significance [64].

## 3. Discussion

The *T_g_* was determined using DSC and the results are summarized in Table 5. The addition of HNTs did not pose any drastic impacts on the *T_g_* of epoxy. For E/HNTs systems, the minor decrease in *T_g_* was attributed to the weak interaction between the HNTs surface and the epoxy molecules. For modified systems, the increase in *T_g_* was observed, but the enhancement could be expected if the interaction between the polymer and nanoparticles was strong enough to restrict the mobility of the matrix. Such a restricted molecular movement could principally be overcome by the increase in temperature. For E/HNT-CAP-L, the *T_g_* was measured as 101.9 °C, which was higher than that for a system containing pristine HNT and other nanocomposites. Aniline pentamer chains acting as a surface activator on HNTs obviously enhanced the accessibility of functional groups participating in the epoxy ring-opening reaction, as flagged by the *Excellent* cure state. Moreover, the strong hydrogen bonding induced by the 8-HQ corrosion inhibitor encapsulated the polar molecules of HNT and hindered the movements of molecules in the nanocomposite, which increased the *T_g_*.

## 4. Materials and Methods

### 4.1. Materials

Ultrafine grade HNTs were purchased from Imerys Tableware Asia Limited (New Zealand) with an internal diameter of 15–20 nm and an external diameter of 50-60 nm, with the composition of SiO_2_ = 49.5 wt%; Al_2_O_3_ = 35.5 wt%; Fe_2_O_3_ = 0.29 wt%; and TiO_2_ = 0.09 wt% [65]. The bulk density and specific area of this white powder were 2.55 g/mL and 65 m^2^/g, respectively. Araldite^®^ LY 5052 epoxy resin with a viscosity of 1000–1500 MPa·s and an epoxide value of 6.65–6.85 Eq./kg, as well as Aradure HY 5052 cross-linking agent, and a mixture of isophorone diamine and cycloaliphatic diamine with a viscosity of 40–60 MPa·s were purchased from Huntsman (USA). Moreover, 8-HQ, succinic anhydride, aniline monomer, N,N-dimethylformamide (DMF), ammonium peroxodisulfate (APS), and 1,4-phenylene diamine were provided by Merck (Darmstadt, Germany). 4-Aminodiphenyl amine and APTES were obtained from Sigma-Aldrich (Darmstadt, Germany).

### 4.2. Synthesis of Aniline Oligomer 

#### 4.2.1. Carboxyl-Capped Aniline Trimer (CAT)

For the synthesis of CAT, 1.86 g aniline monomer (0.02 mol), after purification by steam distillation at 120 °C, and 1.08 g 1,4-phenylene diamine (0.01 mol) were separately dissolved in 50 mL of DMF [66]. Then, the two solutions were mixed, and the reaction system was cooled down to 0 °C by an ice bath. To initiate the chemical reaction, a solution of 0.02 mol APS ((NH_4_)_2_S_2_O_8_) in 100 mL 1 M HCl was added dropwise into the above solution with strong stirring for 30 min. The resulting solution was stirred constantly for 24 h at 0 °C. It was then precipitated into distilled water and washed several times until the pH was neutral. After drying at 50 °C for 24 h, the crude aniline trimer was obtained as a somber green powder.

**Figure 13:**
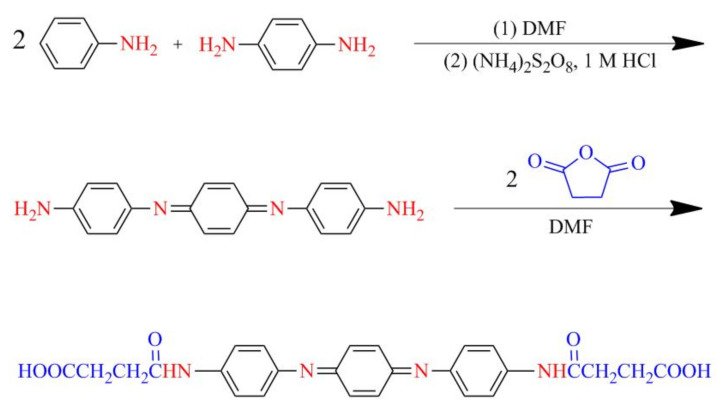
Chemical synthesis pathway of carboxyl-capped aniline trimer.

In order to increase the reactivity of aniline trimer, carboxylic functional groups were attached to both ends through the following procedure. First, 0.01 mol of the synthesized aniline trimer (with amine end-groups) and 0.02 mol succinic anhydride were dissolved in 100 mL DMF. The solution was held at 40 °C under magnetic stirring for 24 h. Then, the reaction mixture was treated by centrifugation at 10,000 rpm, and the precipitate was resuspended in distilled water and centrifuged again; this procedure was repeated at least three times. Finally, CAT was dried in a vacuum oven at 50 °C for 24 h. The synthesis pathway is shown in Figure 13.

#### 4.2.2. Carboxyl-Capped Aniline Pentamer (CAP)

**Figure 14:**
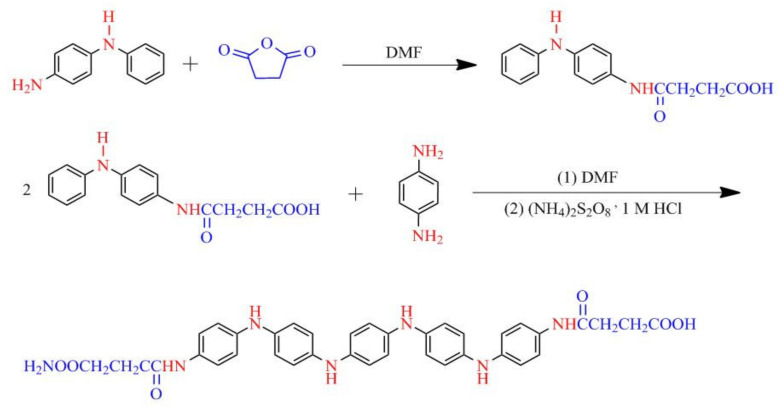
The synthesis pathway of carboxyl-capped aniline pentamer.

For the synthesis of carboxyl-capped aniline pentamer, the procedure described by Hu et al. was employed [67]. In total, 1.84 g 4-aminodiphenylamine (0.01 mol) and 1 g succinic anhydride (0.01 mol) was dissolved in 100 mL DMF and the mixture was stirred for 24 h at 40 °C to provide a succinic acid-protected aniline dimer. The black crude product was obtained after several centrifugations at 10,000 rpm and 24 h drying in a vacuum oven at 50 °C. Subsequently, 0.02 mol of the synthesized dimer was completely dissolved in 100 mL DMF and 0.01 mol 1,4-phenylene diamine was added to the mixture and placed in an ice bath. After the dropwise addition of APS (0.02 mol in 100 mL 1 M HCl), the reaction mixture was held at 0 °C for 24 h and then washed with an excessive amount of distilled water and centrifugation at 10,000 rpm. The precipitation was dried at 50 °C for 24 h, which resulted in a dark green powder (CAP). The synthesis of the CAP is demonstrated in Figure 14.

### 4.3. Preparation of Surface Modified HNTs

#### 4.3.1. Surface Functionalization of HNTs with APTES (A-HNT)

In order to improve the surface reactivity of HNTs for functionalization with oligoanilines, APTES coupling agent was used in the first step. Pristine HNTs were dried at 105 °C for 1 h before modification, owing to its hygroscopic nature. Nanoparticles (HNT, 1 g) were dispersed in 70 mL of dry toluene under sonication for 5 min. Then, 2 mL APTES coupling agent (HNT:APTES ratio (w/v) = 1:2) was added under continuous stirring and refluxed at 80 °C for 48 h. The solid nanotubes were separated centrifugally and washed by ethanol and toluene three times. Lastly, the APTES-modified HNTs (A-HNTs) were dried under vacuum for 24 h at 80 °C. In the second step, the surface-activated HNTs were used for further modification with oligoanilines.

#### 4.3.2. Surface Modification of A-HNTs with Aniline Oligomers (CAT-HNT and CAP-HNT)

At this stage, 1 g of previously synthesized aniline oligomers was separately dissolved in 100 mL of DMF, and 1 mg of A-HNTs was added to each solution under vigorous stirring and at 50 °C. The reaction was continuously carried out for 48 h. Then, the resulting product was washed with DMF several times and dried in a vacuum oven at 50 °C for 24 h.

### 4.4. Encapsulation of Corrosion Inhibitor in Oligoaniline-Modified HNTs

The encapsulation of the corrosion inhibitor 8-HQ in the lumen of prepared HNTs was carried out according to work reported by Shchukin et al. [68]. First, HNTs were mixed with a saturated solution of 8-HQ in ethanol. Then, a sealed beaker containing the suspension was sonicated for 10 min in order to assure the fine dispersion of nanotubes in the solution. The air of the inner side of the nanoparticles was evacuated using a vacuum pump. The slight fizzing of the suspension was indicative of air removal and, consequently, the diffusion of 8-HQ into the lumen of the nanotubes. The suspension was kept under vacuum and continuous stirring for 24 h. During this time, the system was cycled back to atmospheric pressure several times in order to increase the loading efficiency. Finally, 8-HQ-loaded HNT was washed several times with ethanol to remove any physically adsorbed 8-HQ, then collected through centrifugation and drying at 50 °C overnight. This loading procedure was separately conducted on the pristine and surface modified HNTs.

### 4.5. Preparation of Oligoaniline-Modified HNT/Epoxy Nanocomposites

To study the contribution of prepared nanoparticles in the cure behavior of the epoxy system, pristine and 8-HQ-loaded HNTs, CAT-HNTs, and CAP-HNTs were dispersed in epoxy resin in a concentration of 0.1 wt.% resin under sonication with an ultrasound amplitude wave of 50% for 10 min (Branson 8510, Emerson Electric Co., USA). The homogenous nanocomposite mixture was carefully mixed with 100:38 stoichiometric amount of cross-linking agent (Aradure HY 5052) at room temperature and the consequent product was used for the calorimetric cure evaluation study. The nanocomposites were labeled according to the incorporated nanomaterials: E/HNT, E/HNT-L, E/CAT-HNT, E/CAT-HNT-L, E/CAP-HNT, and E/CAP-HNT-L, where the letter “L” is representative of 8-HQ loaded nanoparticles.

### 4.6. Characterization

An FTIR instrument (Spectrum II, PerkinElmer Inc., USA) was used to probe into the chemical structure of the synthesized oligoanilines (CAT and CAP) and the surface modification of the halloysite nanotubes at different stages. FTIR spectra were obtained by using a KBr pellet in a transmission mode within the wavelength of 4000–400 cm^−1^. ^1^H-NMR spectra were collected on a Varian–INOVA 500MHz NMR spectrometer in DMSO-d6 as the solvent at room temperature. This analysis was carried out in order to further characterize the chemical structure of synthesized aniline trimer and pentamer. TGA thermograms of the pristine and oligoaniline-modified HNTs were recorded on a thermogravimetric analyzer (STA6000, PerkinElmer Inc., Norwalk, CT, USA). The purpose of this analysis was to estimate the grafting ratio as well as the thermal stability assessment of the synthesized nanoparticles. To do so, samples of about 10 mg were placed in the sample holder and heated from 20 to 600 °C at the heating rate of 10 °C·min^−1^ under a nitrogen flow rate of 20 mL·min^−1^. UV-visible spectra were measured on a PerkinElmer Lambda 25 spectrophotometer at room temperature. TEM imaging was conducted on a Philips CM100 apparatus (Netherlands) under 100 kV voltage. Furthermore, the microstructures and surface morphologies of HNTs and their prepared epoxy nanocomposites were examined through scanning electron microscopy (SEM) (Mira 3, TESCAN, Brno, Czech Republic) after being coated with gold by using gold sputter coater (Bio-Rad, Watford, England). Furthermore, the SEM was equipped with an energy dispersive X-ray spectrometer (EDS) to investigate the composition and distribution of elements in HNTs and the cross-section of nanocomposite sample free films. SEM micrographs were provided from the cryogenically fractured cross-sectional surface of the fully cured samples. To assess the curing potential of prepared nanomaterials towards epoxy coatings, DSC analysis was conducted via DSC4000 instrument (PerkinElmer Inc., Waltham, MA, USA). For this purpose, samples of about 12 mg were placed in aluminum pans and directly exposed to heating at different rates of 5, 10, 15, and 20 °C·min^−1^ from 15 to 350 °C under nitrogen purging. This heating sweep was followed by a cooling–reheating cycle at the heating rate of 20 °C·min^−1^ to determine T_g_ as an evaluator of final nanocomposite performance.

## 5. Conclusions

This project successfully introduced novel nanocontainers possessing halloysite nanotubes acting as the core and oligoanilines with two different lengths (CAT and CAP) acting as the shell of the nanomaterial. The aniline trimer and pentamer chains were deposited onto the surface of HNTs through APTES coupling agents. The synthesis and grafting processes used in this study were verified by FTIR, H-NMR, TGA, and SEM analyses. The empty spaces within the prepared nanocontainer systems were loaded with 8-HQ corrosion inhibitor and confirmed by UV-visible spectroscopy. The final loaded nanocontainers were incorporated into the epoxy resin to study their contribution in cure kinetics and the behavior of epoxy/amine systems. The thermal variations of the nanocomposite system during network formation were recorded through nonisothermal DSC measurements at four different heating rates. The isoconversional methods of Friedman and KAS were applied to calculate the cure apparent activation energies at various conversions. Moreover, the reaction model was chosen based on the Malek method, which resulted in the selection of the Sestak–Berggren model for the autocatalytic cross-linking reactions. It was observed that although HNTs restricted the accessibility of cure moieties compared to a blank resin, the incorporation of oligoaniline-modified HNTs could increase the heat of reaction compared to the unmodified epoxy system by more than 8% and 17% at the heating rate of 5 °C/min for the nanotubes with CAT and CAP shells, respectively. Moreover, for almost all the systems containing aniline oligomer shells, the curing temperature window was narrowed, which shows the facilitating role of oligoanilines containing amine functional groups. The entrapment of 8-HQ in the prepared nanocontainers led to a higher enthalpy of reaction (up to 30% for E/CAT-HNT-L at the heating rate of 5 °C/min) while extending the temperature range in which the reaction occurs. However, a higher length of shell molecules in CAP-modified HNTs induced greater encapsulation efficiency, and thus, not only did the 8-HQ loading process narrow the temperature window, but it also decreased it to values lower than that of systems containing unloaded CAP-HNT nanomaterials. Moreover, the CAP shell intensified the autocatalytic reactions occurring in the system. As a result, the reactivity and chain length of polymeric shells and the loading efficiency of the nanoparticles and their dispersion state in the epoxy matrix were mentioned as the most important factors to be considered for designing an optimal multifunctional system. Finally, the suggested theoretical models predicted the experimental data with a good approximation.

## Figures and Tables

**Table 1:** Cure characteristics of epoxy and its nanocomposites as a function of heating rate (*β*).

Designation	*β* (°C/min)	*T_onset_* (°C)	*T_p_* (°C)	*T_endset_* (°C)	*ΔT* (°C)	*ΔH_∞_* (J/g)	ΔT^*^ (°C)	ΔH^*^_∞_ (J/g)	*CI*	Quality
Epoxy	5	42.1	102.7	199.9	157.9	406.0	-	-	-	-
	10	55.3	117.6	209.8	154.5	388.2	-	-	-	-
	15	57.3	125.6	218.5	161.3	402.9	-	-	-	-
	20	56.3	132.2	246.0	189.7	471.7	-	-	-	-
E/HNT	5	46.1	99.7	190.8	144.8	348.3	0.92	0.86	0.79	*Poor*
	10	50.4	115.6	199.9	149.5	365.5	0.97	0.94	0.91	*Poor*
	15	56.7	125.6	215.5	158.8	395.2	0.99	0.98	0.97	*Poor*
	20	59.2	133.8	231.5	172.4	376.6	0.91	0.80	0.73	*Poor*
E/CAT-HNT	5	45.9	103.6	177.4	131.5	437.9	0.83	1.08	0.90	*Excellent*
	10	54.3	117.0	208.7	154.4	425.5	1.00	1.10	1.10	*Excellent*
	15	59.2	125.8	207.9	148.8	400.4	0.92	0.99	0.92	*Poor*
	20	68.2	130.7	215.8	147.7	373.3	0.78	0.79	0.62	*Poor*
E/CAP-HNT	5	47.7	103.8	179.9	132.3	476.6	0.84	1.17	0.98	*Excellent*
	10	51.4	118.4	195.6	144.2	418.7	0.93	1.08	1.01	*Excellent*
	15	61.7	127.1	227.0	165.3	397.6	1.03	0.99	1.01	*Poor*
	20	65.5	131.2	230.9	165.4	415.8	0.87	0.88	0.77	*Poor*
E/HNT-L	5	40.8	105.1	201.4	160.5	492.1	1.02	1.21	1.23	*Good*
	10	52.1	117.8	217.7	165.6	500.2	1.07	1.29	1.38	*Good*
	15	53.2	125.3	231.2	178.0	448.5	1.10	1.11	1.23	*Good*
	20	59.8	133.2	222.8	163.0	379.3	0.86	0.80	0.69	*Poor*
E/CAT-HNT-L	5	33.1	103.4	208.9	175.9	526.1	1.11	1.30	1.44	*Good*
	10	39.44	115.1	233.3	193.9	454.6	1.26	1.17	1.47	*Good*
	15	41.5	123.8	244.6	203.0	578.2	1.26	1.44	1.81	*Good*
	20	50.5	132.2	273.9	223.4	426.5	1.18	0.90	1.07	*Poor*
E/CAP-HNT-L	5	50.2	105.0	169.3	119.1	409.6	0.76	1.01	0.76	*Excellent*
	10	54.3	114.7	186.8	132.5	395.2	0.86	1.02	0.88	*Excellent*
	15	61.4	124.1	214.7	153.3	451.3	0.95	1.12	1.07	*Excellent*
	20	69.2	131.5	219.6	150.4	396.6	0.79	0.84	0.67	*Poor*

**Table 2:** The values of *α_p_*, *α_m_*, and *α_p_^∞^* obtained from DSC analysis based on Malek model at various heating rates (*β*).

Designation	*β* (°C/min)	*α_p_^∞^*	*α_m_*	*α_p_*
Epoxy	5	0.449	0.058	0.484
	10	0.498	0.080	0.489
	15	0.546	0.080	0.501
	20	0.488	0.087	0.490
E/HNT	5	0.440	0.096	0.480
	10	0.586	0.108	0.504
	15	0.576	0.135	0.501
	20	0.581	0.152	0.502
E/CAT-HNT	5	0.488	0.071	0.526
	10	0.414	0.060	0.500
	15	0.510	0.104	0.518
	20	0.454	0.107	0.511
E/CAP-HNT	5	0.460	0.091	0.525
	10	0.554	0.102	0.534
	15	0.394	0.091	0.488
	20	0.398	0.101	0.493
E/HNT-L	5	0.470	0.072	0.512
	10	0.460	0.061	0.491
	15	0.483	0.056	0.492
	20	0.599	0.104	0.501
E/CAT-HNT-L	5	0.452	0.041	0.497
	10	0.425	0.065	0.484
	15	0.467	0.093	0.501
	20	0.358	0.089	0.483
E/CAP-HNT-L	5	0.505	0.098	0.541
	10	0.496	0.036	0.552
	15	0.392	0.081	0.519
	20	0.403	0.100	0.513

**Table 3:** Kinetic parameters of the studied systems obtained from Friedman model at different heating rates.

Designation	*β* (°C/min)	*Ē_a_* (kJ/mol)	*ln A* (s^−1^)	Mean Value (s^−1^)	*m*	Mean Value	*n*	Mean Value
Epoxy	5	55.01	16.18	16.22	0.10	0.11	1.79	1.79
	10		16.12		0.07		1.73	
	15		16.27		0.12		1.74	
	20		16.30		0.17		1.90	
E/HNT	5	48.99	14.30	14.40	0.13	0.18	1.61	1.64
	10		14.36		0.14		1.57	
	15		14.46		0.20		1.66	
	20		14.48		0.24		1.70	
E/CAT-HNT	5	54.06	15.98	16.03	0.16	0.17	1.58	1.66
	10		15.94		0.12		1.74	
	15		16.07		0.21		1.67	
	20		16.11		0.21		1.67	
E/CAP-HNT	5	47.67	13.94	14.03	0.26	0.25	1.54	1.62
	10		14.09		0.28		1.55	
	15		13.98		0.21		1.70	
	20		14.12		0.25		1.69	
E/HNT-L	5	58.64	17.49	17.39	0.18	0.13	1.89	1.86
	10		17.35		0.10		1.88	
	15		17.37		0.09		1.89	
	20		17.36		0.14		1.77	
E/CAT-HNT-L	5	53.10	15.67	15.74	0.18	0.19	1.86	1.89
	10		15.71		0.13		1.88	
	15		15.85		0.24		1.87	
	20		15.72		0.20		1.93	
E/CAP-HNT-L	5	47.60	13.97	14.12	0.28	0.28	1.43	1.51
	10		14.13		0.25		1.39	
	15		14.18		0.28		1.62	
	20		14.20		0.30		1.60	

**Table 4:** Kinetic parameters of the studied system obtained from KAS model at different heating rates.

Designation	*β* (°C/min)	*Ē_a_* (kJ/mol)	*ln A (s*^−1^)	Mean Values (s^−1^)	*m*	Mean Values	*n*	Mean Values
Epoxy	5	54.67	16.08	16.11	0.10	0.12	1.79	1.79
	10		16.01		0.08		1.73	
	15		16.16		0.13		1.74	
	20		16.20		0.17		1.89	
E/HNT	5	48.26	14.06	14.17	0.13	0.19	1.60	1.63
	10		14.14		0.15		1.56	
	15		14.23		0.21		1.65	
	20		14.26		0.25		1.69	
E/CAT-HNT	5	52.73	15.56	15.62	0.17	0.19	1.57	1.65
	10		15.53		0.14		1.72	
	15		15.67		0.22		1.65	
	20		15.72		0.22		1.65	
E/CAP-HNT	5	51.35	15.10	15.16	0.22	0.21	1.59	1.67
	10		15.22		0.24		1.60	
	15		15.09		0.17		1.75	
	20		15.21		0.21		1.74	
E/HNT-L	5	57.06	16.99	16.91	0.20	0.15	1.87	1.84
	10		16.87		0.11		1.86	
	15		16.89		0.11		1.87	
	20		16.90		0.16		1.75	
E/CAT-HNT-L	5	53.44	15.78	15.84	0.18	0.18	1.87	1.89
	10		15.82		0.13		1.89	
	15		15.96		0.23		1.87	
	20		15.82		0.19		1.94	
E/CAP-HNT-L	5	50.24	14.81	14.93	0.25	0.25	1.46	1.54
	10		14.94		0.22		1.42	
	15		14.98		0.25		1.65	
	20		14.98		0.27		1.63	

**Table 5:** The values of *T_g_* measured for the neat epoxy and its nanocomposites.

Sample	*T_g_* (°C)
Neat epoxy	97.5
E/HNT	97.3
E/HNT-L	98.1
E/HNT-CAT	99.2
E/HNT-CAP	99.8
E/HNT-CAT-L	100.3
E/HNT-CAP-L	101.9

**Figure A7:**
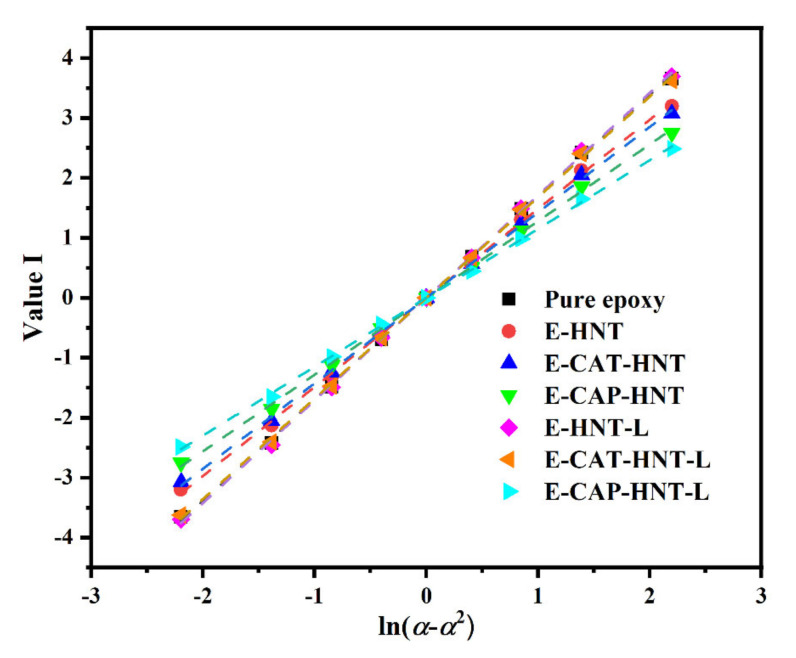
Plots of Value I calculated using DSC data for prepared epoxy system under heating rate of 5 °C/min.

**Figure A8:**
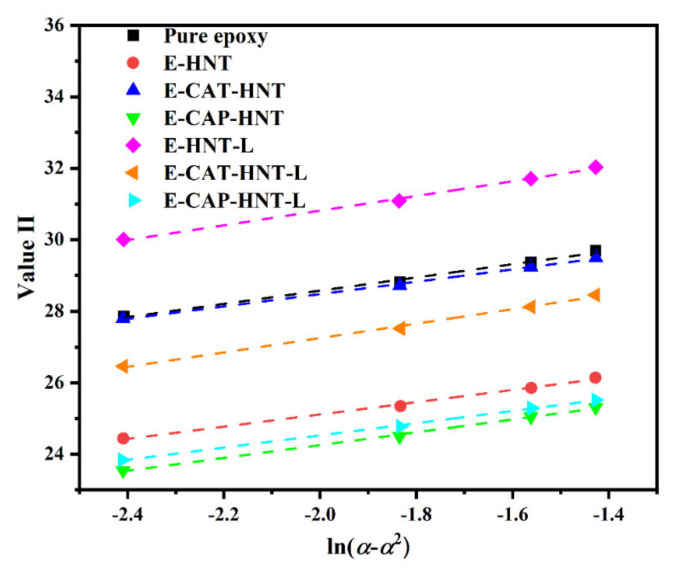
Plots of Value II calculated for prepared samples under heating rate of 5 °C/min.

## Data Availability

Data available as per request from corresponding authors.

## Appendix B. Isoconversional Kinetic Methods

In nonisothermal DSC, the rate of reaction at every heating rate is proportional to the rate of heat generation and is defined as below:

(*dα*/*dt*) = *k*(*T*) × *f*(*α*) (A1)

where *f*(*α*) is the reaction model and *k*(*T*) shows the reaction constant, simply parameterized by Arrhenius equation, which specifies the Equation (A1) as below:

(*dα*/*dt*) = *A* × exp(−*E_α_*/*RT*) × *f*(*α*) (A2)

where *E_a_*, *A*, and *R* account for activation energy, frequency factor of collision, and universal gas constant, respectively. In isoconversional model-free methods, it is assumed that the reaction rate at a specific conversion is merely a function of temperature.

### Appendix B.1. Friedman Model

By a rearrangement of Equation (A4), Friedman model is obtained as the following equation:

ln(*β_I_* × (*dα*/*dt*)*_α,i_*) = ln(*f*(*α*) × *A_α_*) − (*E_α_*/(*RT_α,i_*)) (A3)

where *α*, *β*, and *E_α_* are representative of cure conversion, heating rate, and activation energy, respectively. Depicting this vs. 1/*Tα*, one can find the values of activation energy at each conversion from the slope of Figure A2.

### Appendix B.2. KAS Method

KAS method is defined by the following equation:

ln(*β_I_*/*T_α,i_*^1.92^) = const. − 1.0008 × *E_α_*/(*RT_α_*) (A4)

Plotting vs. 1/*Tα* provides the possibility of obtaining the apparent activation energy from the slope of straight lines shown in Figure A3.

## Appendix C. Determination of Curing Reaction Model

### Appendix C.1. Friedman Model

Based on the Friedman method, the model of epoxy curing reaction can be determined using Equation (A5). The shape of the plot of ln[Af(*α*)] vs. ln(1 − *α*) represents the deviation from the nth order reaction (Figure A4).

ln(*A* × *f*(*α*)) = ln(*dα*/*dt*) + (*E*/*RT*) = ln*A* + *n* × ln(1 − *α*) (A5)

For nth-order cure kinetics a straight line should be obtained by plotting ln[Af(*α*)] vs. ln(1 − *α*) wherein slope provides the reaction rate (*n*).

According to the Friedman method, based on the shape of the plot of ln[Af(α)] against ln(1 − *α*), the curing reaction can be determined (Equation (A5)).

### Appendix C.2. Malek Method

The important parameters of Malek method *y*(*α*) and *z*(*α*) can be determined by Equations (A6) and (A7) as follows:

*y*(*α*) = (*dα*/*dt*)*_α_* × exp(*E*_0_/(*RT_α_*) = *A* × *f*(*α*) (A6)

*z*(*α*) = (*dα*/*dt*)*_α_* × *T_α_*^2^ × *π*(*χ*)/*βT_α_* (A7)

The term in the brackets of Equation (A7) is negligible and ineffective on the shape of the *z*(*α*) function, and therefore can be omitted. In Equation (A6), the first trial for *E*_0_ is determined by isoconversional methods, for instance the Flynn-Wall-Ozawa (FWO) model (Equation A4), in which the apparent activation energy is determined from the slope of ln(*β_i_*) vs. 1000/*T_p_* plot, as shown in Figure A5. The experimental values of *y*(*α*) and *z*(*α*) for the epoxy nanocomposite as a function of α are shown in Figure A6 and compared with theoretical master plots.

ln(*β_i_*) = const. − 1.052 × *E_α_*/(*RT_α_*) (A8)

The values of y(*α*) and z(*α*) should be normalized as follows to take a value between 0 and 1:

*y_n_*(*α*) = (*y*(*α*)/*dt*)*_α_* × exp(*E*_0_/(*RT_α_*) = *A* × *f*(*α*) (A9)

*z_n_*(*α*) = (*dα*/*dt*)*_α_* × *T_α_*^2^ × *π*(*χ*)/*βT_α_* (A10)

The maximum values of *y*(*α*)max = *α*m and *z*(*α*)max = *α*_p_ can be deduced from the following expressions, which are critical for kinetic characterization:

*f’*(*α_m_*) = 0
(A11)

*f’*(*α_p_*) × *g*(*α_p_*) = −1
(A12)

The parameter *g*(*α*_p_) is the integral form of the reaction model. The results of calculations are reported in Table 2.

## Appendix D. Determination of the Degree of Reaction

The degrees of autocatalytic reaction (n and m) and the frequency factor (A) as the essential kinetic parameters can be determined through the following equations:

*Value I* = ln(*dα*/*dt*) + (*E_α_*/*RT*) − ln((*d* × (1 − *α*))/*dt*) − (*E_α_*/*RT’*) = (*n* − *m*) × ln((1 − *α*)/*α*) (A13)

*Value II* = ln(*dα*/*dt*) + (*E_α_*/*RT*) + ln((*d* × (1 − *α*))/*dt*) + (*E_α_*/*RT’*) = (*n* + *m*) × ln(*α* − *α*^2^) + 2ln*A* (A14)

The slope of the plots of *Value I* vs. ln[(1 − *α*)/*α*] (Figure A7) and *Value II* vs. ln(*α* − *α*^2^) (Figure A8) provides the value of (*n* − *m*) and (*n* + *m*), respectively, and from the intercept of the later plot, the A value can be calculated. The results are reported in Table 3 and Table 4.
